# Elucidating molecular interactions of *L*-nucleotides with HIV-1 reverse transcriptase and mechanism of M184V-caused drug resistance

**DOI:** 10.1038/s42003-019-0706-x

**Published:** 2019-12-13

**Authors:** Magdeleine Hung, E. John Tokarsky, Leanna Lagpacan, Lijun Zhang, Zucai Suo, Eric B. Lansdon

**Affiliations:** 1 Gilead Sciences, Inc., 333 Lakeside Dr., Foster City, CA 94404 USA; 2 The Ohio State Biophysics Program, The Ohio State University, Columbus, OH 43210 USA; 3 Department of Biomedical Sciences, Florida State University College of Medicine, Tallahassee, FL 32306 USA

**Keywords:** Enzyme mechanisms, HIV infections, X-ray crystallography, Pharmaceutics

## Abstract

Emtricitabine (FTC) and lamivudine (3TC), containing an oxathiolane ring with unnatural (−)-stereochemistry, are widely used nucleoside reverse transcriptase inhibitors (NRTIs) in anti-HIV therapy. Treatment with FTC or 3TC primarily selects for the HIV-1 RT M184V/I resistance mutations. Here we provide a comprehensive kinetic and structural basis for inhibiting HIV-1 RT by (−)-FTC-TP and (−)-3TC-TP and drug resistance by M184V. (−)-FTC-TP and (−)-3TC-TP have higher binding affinities (1/*K*_d_) for wild-type RT but slower incorporation rates than dCTP. HIV-1 RT ternary crystal structures with (−)-FTC-TP and (−)-3TC-TP corroborate kinetic results demonstrating that their oxathiolane sulfur orients toward the DNA primer 3′-terminus and their triphosphate exists in two different binding conformations. M184V RT displays greater (>200-fold) *K*_d_ for the *L*-nucleotides and moderately higher (>9-fold) *K*_*d*_ for the *D*-isomers compared to dCTP. The M184V RT structure illustrates how the mutation repositions the oxathiolane of (−)-FTC-TP and shifts its triphosphate into a non-productive conformation.

## Abstract (web summary)

Magdeleine Hung et al. report the comprehensive kinetic and structural basis for the inhibition of HIV-1 Reverse Transcriptase by FTC and 3TC, as well as the drug resistant M184V variant. Structural analysis of HIV-1 RT and inhibitors provide insights into the mechanism of binding and resistance.

## Introduction

**Fig. 1:**
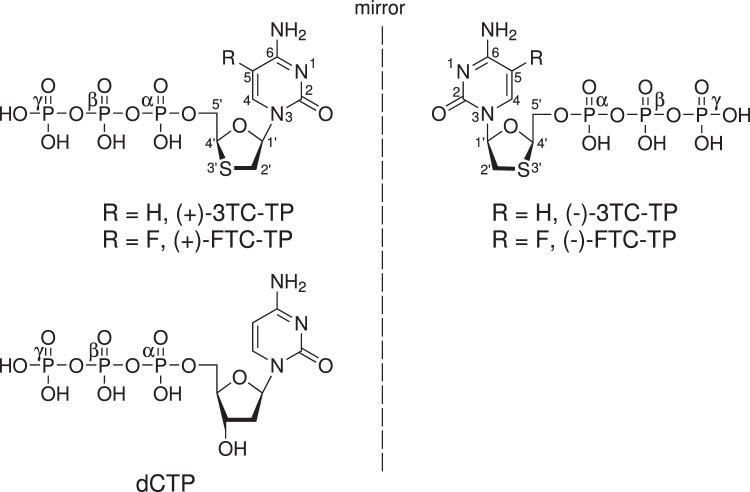
Chemical structures of (−)-FTC-TP, (+)-FTC-TP, (−)-3TC-TP, (+)-3TC-TP, and the natural substrate deoxycytidine triphosphate (dCTP). The stereochemistry of the oxathiolane ring has an unnatural *L*-conformation in (−)-FTC-TP and (−)-3TC-TP as opposed to the natural *D*-conformation found with dCTP, (+)-3TC-TP, and (+)-FTC-TP.

Human immunodeficiency virus (HIV) and acquired immunodeficiency syndrome (AIDS) afflict approximately 36.9 million people worldwide according to the WHO^1^. The most effective treatment regimen is highly active antiretroviral therapy (HAART) which consists of a backbone of nucleoside or nucleotide reverse transcriptase inhibitors (NRTIs) combined with either a protease inhibitor, a non-nucleoside reverse transcriptase inhibitor (NNRTI), or an integrase strand transfer inhibitor (INSTI). Of the available NRTIs as treatment options, tenofovir disoproxil fumarate (TDF) and emtricitabine (FTC) (Fig. 1) are part of the recommended treatment backbone^2^. These two drugs are available in a single pill (Truvada®) which in addition to being used in HIV therapy is approved for pre-exposure prophylaxis to prevent HIV infection. Single tablet regimens combining TDF and FTC with an additional active anti-HIV agent are also available (Atripla®, Complera®, and Stribild®). These treatment options allow patients to take just one pill a day to control their HIV infection. Recently, FTC has been combined with tenofovir alafenamide, a next generation prodrug of tenofovir approved for HIV treatment (Descovy®), along with elvitegravir and cobicistat (Genvoya®) as well as rilpivirine (Odefsey®).

NRTIs mimic natural deoxynucleosides or deoxynucleotides and target the reverse transcriptase (RT) enzyme. RT incorporates active metabolites of NRTIs into the growing DNA chain which act as chain terminators due to their lack of 3ʹ-OH. The NRTIs FTC (2ʹ,3ʹ-dideoxy-5-fluoro-3ʹ-thiacytidine) and 3TC (lamivudine; 2ʹ,3ʹ-dideoxy-3ʹ-thiacytidine) are cytidine analogs. FTC and 3TC are both administered in their 5ʹ-OH form and once absorbed into cells are converted by cellular kinases into their active metabolites FTC-triphosphate ((−)-FTC-TP) and 3TC-triphosphate ((−)-3TC-TP), respectively^3^. The triphosphate form is recognized by HIV-1 RT and is incorporated into the DNA primer strand, leading to the termination of viral replication. In initial cell-based screening assays, FTC was found to be more potent and less cytotoxic than its corresponding (+)-stereoisomer against the HIV virus, thus leading to further development of the (−)-enantiomer^3^. Most notably, in place of the deoxyribose ring, these drugs contain an oxathiolane ring in a (−)-β-*L* configuration, with  the opposite stereochemistry from normal *D*-ribose (Fig. 1). In clinical trials as a monotherapy, FTC demonstrated a 1.7 log reduction in viral HIV RNA^4^. In addition to being approved to treat HIV, FTC and 3TC are also effective inhibitors of hepatitis B virus (HBV) polymerase. Currently, 3TC is an approved antiviral drug for HBV treatment and FTC has been tested in clinical trials^5^.

Both in vitro and in vivo experiments have shown that FTC and 3TC primarily select for resistance mutations, M184V or M184I^6–8^. M184 is part of the conserved active site YMDD motif found in several viral polymerases, including HIV-1 RT and HBV polymerase. The M184I mutation will often emerge first, possibly because it results from a single nucleotide change^9^. M184V arises from a two nucleotide change but it outcompetes M184I and is observed in most patients with virologic failure resulting from FTC or 3TC treatment^8,9^. The M184V mutation has been shown to have multiple effects on RT activity and resistance^10^. As a single amino acid residue mutation, M184V confers very high resistance to FTC and 3TC as compared to WT (>500-fold)^6^. The mutation has been associated with lower polymerase processivity; however, it confers higher fidelity for correct dNTPs^11–13^ and is connected with reduced viral replication^14^. While M184V provides resistance to FTC and 3TC, it can concurrently lead to an increased susceptibility to TDF, stavudine, and azidothymidine (AZT), and can slow the resistance development against these NRTIs^15^.

The mechanism of M184V resistance to FTC and 3TC has been debated in the literature. Several groups have explored whether M184V confers NRTI resistance by reducing binding affinities of these *L*-nucleotide analogs by the HIV-1 RT or whether it affects the rates of *L*-nucleotide analog incorporation. Steady-state and pre-steady-state kinetic data collected by Krebs et al. suggested that the M184V mutation primarily affects the rate of incorporation of the *L*-nucleotide analogs^16^. Additionally, Gao et al. using gel shift assays, demonstrated reduced rates of *L*-nucleotide analog incorporation by the M184V mutant with no effect on their binding^17^. On the other hand, kinetic experiments from Wilson et al. and Feng et al. suggest that the mutation primarily affects *L*-nucleotide analog binding to HIV-1 RT^18,19^.

Crystal structures of (−)-FTC-TP and (−)-3TC-TP have been reported with various DNA polymerases^20,21^ but not HIV-1 RT. We sought to establish the features of WT HIV-1 RT that recognize the unique stereochemistry of (−)-β-*L* oxathiolane analogs. Herein, using the same DNA construct, we report the pre-steady-state kinetic analysis with WT RT and M184V to determine the incorporation efficiency of both (+)- and (−)-enantiomers of FTC-TP and 3TC-TP compared to dCTP in addition to crystal structures of WT RT in complex with dsDNA (RT–DNA) bound to either, (−)-FTC-TP, (−)-3TC-TP, (+)-FTC-TP, or dCTP. Furthermore, binary (M184V-DNA), and ternary structures with (−)-FTC-TP and dCTP were determined to better understand the mechanism by which the M184V mutation confers resistance to NRTIs. These comprehensive kinetic and structural studies provide the most definitive insights into the binding modes of oxathiolane analogs and the mechanism of NRTI resistance achieved by the M184V mutation of HIV-1 RT.

## Results

### Pre-steady-state kinetics of *L*-nucleotide incorporation

**Fig. 2:**
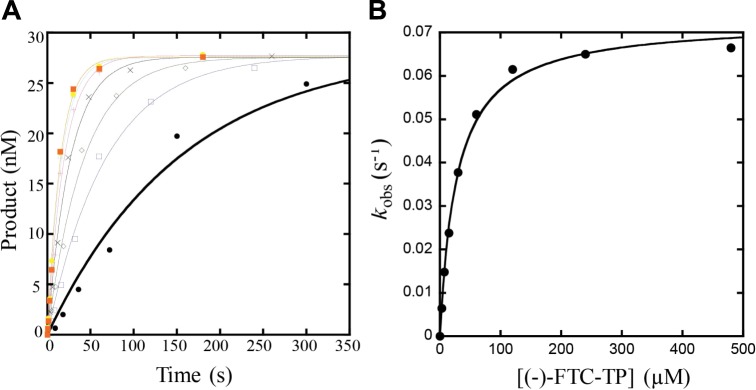
Determination of kinetic parameters for (−)-FTC-TP incorporation onto 18/26-mer dsDNA catalyzed by the M184V mutant of HIV-1 RT. **a** A pre-incubated solution of 120 nM M184V and 30 nM 5′-[^32^P]-labeled 18/26-mer was mixed with increasing concentrations of (−)-FTC-TP (3.75 µM, ●; 7.5 µM, ; 15 µM, ; 30 µM, x; 60 µM, ; 120 µM, ; 240 µM, ; 480 µM, ) for various times at 37 °C before being quenched with 0.37 M EDTA. The DNA product concentrations were plotted against reaction times and each time course was fit to a single-exponential equation (Materials and Methods) to yield *k*_obs_. **b** The *k*_obs_ values were then plotted against respective concentrations of (−)-FTC-TP and the plot was fit to a hyperbolic equation (Materials and Methods) to yield a *k*_p_ of 0.073 ± 0.001 s^−1^ and a *K*_d_ of 28 ± 2 μM (Table 1).

Previous kinetic studies^16,18,20,22–26^ have aimed at understanding the efficiency of incorporation for (−)-FTC-TP or (−)-3TC-TP, how they compete against dCTP, and the effect of resistance mutations, such as M184V. As in our previous work^20,25,26^, single-turnover kinetic assays were performed by rapidly mixing a pre-incubating solution of 120 nM HIV-1 RT (WT or M184V) and 30 nM 5ʹ-[^32^P]-labeled 18/26-mer dsDNA substrate (Methods) with varying concentrations of dCTP, or an active metabolite of NRTIs for different times, before being quenched by 0.37 M EDTA. Representative kinetic plots displaying M184V-catalyzed incorporation of (−)-FTC-TP onto 18/26-mer dsDNA substrate are shown in Fig. 2. Notably, the same 18/26-mer dsDNA substrate was used in both the single-turnover kinetic assays and crystal structure determination (see below). We determined the pre-steady-state kinetic parameters of maximal nucleotide incorporation rate constants (*k*_p_) and apparent equilibrium dissociation constants (*K*_d_) for dCTP, (+)-FTC-TP, (−)-FTC-TP, (+)-3TC-TP and (−)-3TC-TP with WT or M184V HIV-1 RT (Table 1). Comparing the measured *K*_d_ values for WT RT, both (−)-FTC-TP (0.10 ± 0.01 µM) and (−)-3TC-TP (0.25 ± 0.01 µM) exhibit substantially tighter binding compared to natural dCTP (6.3 ± 0.2 µM), a 63- and 25-fold increase, respectively. Conversely, the *k*_p_ was 119- and 175-fold slower for (−)-FTC-TP (0.100 ± 0.001 s^−1^) and (−)-3TC-TP (0.068 ± 0.001 s^−1^), respectively, than dCTP (11.9 ± 0.1 s^−1^). The resulting selectivity factors ((*k*_p_/*K*_d_)_dCTP_/(*k*_p_/*K*_d_)_NRTI_) were determined to be only 1.9 and 7.0-fold higher for dCTP over (−)-FTC-TP and (−)-3TC-TP, respectively. These data suggest that these chain-terminating *L*-nucleotide analogs are excellent competitive inhibitors as to dCTP during WT HIV-1 RT-catalyzed viral genome replication. Kinetic analysis of the (+)-analogs show similar *K*_d_ values to the (−)-analogs; however, the *k*_p_ values were slightly higher resulting in selectivity factors of 2.4 and 2.5 for (+)-FTC-TP and (+)-3TC-TP, respectively. Furthermore, (−)-FTC-TP (1.0 µM^−1^ s^−1^) was incorporated by WT HIV-1 RT with a 3.7-fold higher efficiency (*k*_p_/*K*_d_) than (−)-3TC-TP (0.27 µM^−1^ s^−1^), a result driven by the 2.5-fold binding affinity increase (Table 1).

**Table 1:** Pre-steady-state kinetic parameters for incorporation of dCTP, FTC-TP, and 3TC-TP by WT or M184V HIV-1 RT at 37 °C.

HIV-1 RT	Nucleotide	*K*_d_ (µM)	*k*_p_ (s^−1^)	*k*_p_/*K*_d_ (µM^−1^ s^−1^)	Selectivity factor^a^
WT	dCTP	6.3 ± 0.2	11.9 ± 0.1	1.9	—
WT	(−)-3TC-TP	0.25 ± 0.01	0.068 ± 0.001	0.27	7.0
WT	(+)-3TC-TP	0.202 ± 0.004	0.26 ± 0.02	0.78	2.5
WT	(−)-FTC-TP	0.10 ± 0.01	0.100 ± 0.001	1.0	1.9
WT	(+)-FTC-TP	0.19 ± 0.02	0.24 ± 0.01	0.79	2.4
M184V	dCTP	8.9 ± 1.0	20.5 ± 0.9	2.3	—
M184V	(−)-3TC-TP	53 ± 4	0.050 ± 0.002	9.4 × 10^−4^	2400
M184V	(+)-3TC-TP	5.4 ± 1.2	0.18 ± 0.01	3.3 × 10^−2^	69
M184V	(−)-FTC-TP	28 ± 2	0.073 ± 0.001	2.6 × 10^−3^	880
M184V	(+)-FTC-TP	1.8 ± 0.3	0.13 ± 0.01	7.6 × 10^−2^	30

^a^(*k*_p_/*K*_d_)_dCTP_/(*k*_p_/*K*_d_)_3TC-TP_ or (*k*_p_/*K*_d_)_dCTP_/(*k*_p_/*K*_d_)_FTC-TP_

As expected, M184V incorporated dCTP (*k*_p_/*K*_d_ = 2.3 µM^−1^ s^−1^) with a similar efficiency to WT (*k*_p_/*K*_d_ = 1.9 µM^−1^ s^−1^) (Table 1). However, the binding affinities of the M184V mutant for (−)-FTC-TP and (−)-3TC-TP were reduced (280-fold and 212-fold, respectively), compared to WT, whereas the *k*_p_ values remain relatively unchanged. Conjointly, the selectivity factors increased to 880 for (−)-FTC-TP and 2400 for (−)-3TC-TP. Interestingly, (+)-FTC-TP (*K*_d_ = 1.8 ± 0.3 µM) and (+)-3TC-TP (*K*_d_ = 5.4 ± 1.2 µM) were bound more tightly to M184V than the (−)-analogs leading to relatively lower selectivity factors of 30 and 69, respectively. Of note, differences in observed kinetic parameters in this study versus previous reports^16,18,19^ may be related to different reaction conditions and dsDNA substrates containing different DNA sequences and ends (blunt-end versus staggered-end).

### *L*-nucleotide structures show two triphosphate conformations

**Fig. 3:**
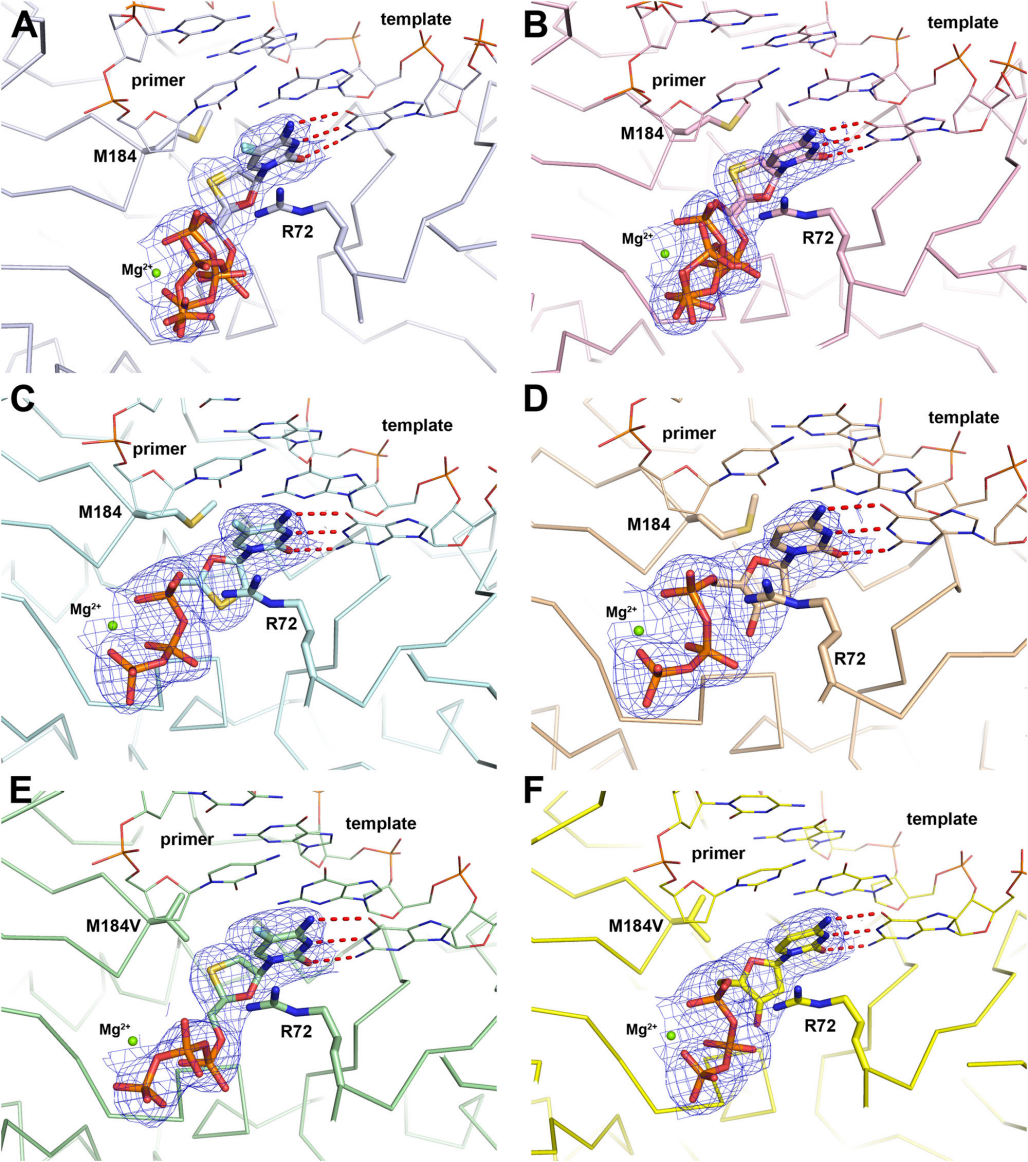
Omit 2Fo-Fc electron maps (blue mesh) for ternary complexes of WT and M184V HIV-1 RT. Ternary complexes of WT and M184V HIV-1 RT showing 2*F*_o_ − *F*_c_ omit electron density maps (blue mesh) of (**a**) RT–DNA•(−)-FTC-TP (PDB code 6UJX), (**b**) RT–DNA•(−)-3TC-TP (PDB code 6UJY), (**c**) RT–DNA•(+)-FTC-TP (PDB code 6UJZ), (**d**) RT–DNA•dCTP (PDB code 6UIT), (**e**) M184V-DNA•(−)-FTC-TP (PDB code 6UIR), and (**f**) M184V-DNA•dCTP (PDB code 6UIS).

**Fig. 4:**
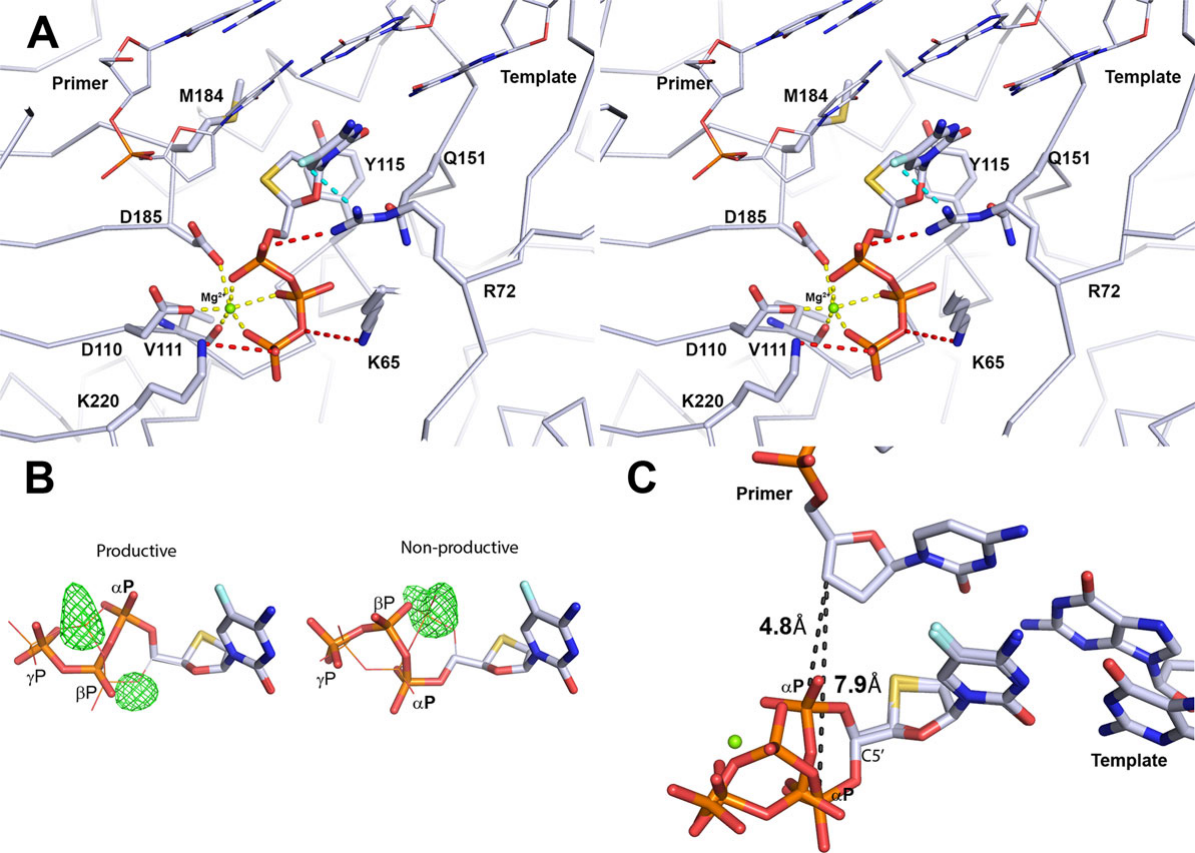
Ternary structure of WT RT–DNA bound to (−)-FTC-TP (light blue). **a** Stereoview of the structure of the ternary RT–DNA•(−)-FTC-TP complex. Only the productive triphosphate binding conformation of (−)-FTC-TP is shown for simplicity. The unnatural (−)-β-*L* oxathiolane ring points back towards the last base in the DNA primer strand. There is a Mg^2+^ ion that forms the typical octahedral coordination (yellow dashed lines) with the triphosphates and active site residues of RT. **b** Fo-Fc map (green mesh) drawn at +4.5σ. Calculated by refining either the productive or non-productive triphosphate conformation independently. The lines show how the alternate conformation fills out the extra e^−^ density. **c** The productive conformation brings the α-phosphate to 4.8 Å (black dashed lines) from the 3ʹ-carbon of the primer 3′-terminal nucleotide while the α-phosphate in the non-productive conformation is 7.9 Å away.

To understand the interaction of cytidine analog drugs with WT HIV-1 RT, crystal structures were determined by covalently cross-linking purified RT to an 18/26-mer dsDNA substrate (Methods) via an *N*^2^-cystamine-deoxyguanosine to Q258C present in the p66 subunit of RT^27^. The ternary complex determined with RT, DNA, and (−)-FTC-TP (RT–DNA•(−)-FTC-TP) displays good electron density for the NRTI and clearly shows the position of the oxathiolane ring and 5-fluoro cytidine base. Normal Watson–Crick base pairing is observed with the template guanine (Fig. 3a). The (−)-β-*L* oxathiolane ring sits above Y115 (Fig. 4a) and faces towards the last nucleobase of the primer strand. The sulfur atom within the ring points towards M184 and is within van der Waals distance to the side chain of M184 (4.0 Å). The phosphates are coordinated by one Mg^2+^ ion and the side chains of R72, K65, and K220 (Fig. 4a). The Mg^2+^ interacts in a typical octahedral coordination with oxygen atoms from all three phosphates as well as the side chains of D110, D185, and the backbone carbonyl of V111. After the initial refinement of the structure it became apparent that the triphosphates were adopting an alternate conformation in addition to the canonical conformation associated with nucleotide binding (Fig. 4b). A second conformation was simultaneously refined where the α-phosphate is flipped down into a nearly identical position as the β-phosphate in the first conformation described above (Fig. 3a). This brings the α-phosphate further away from the primer 3′-terminal nucleotide, likely preventing proper orientation for incorporation into DNA (Fig. 4c). The β-phosphate is flipped up (relative to the first conformation) and the γ-phosphorous atom is in a comparable position in both conformations. We refer to the first as a catalytically competent and productive conformation primed for incorporation and the second conformation as catalytically ineffective, or non-productive. After structure refinement and occupancy optimization, a split of 55%/45% for productive/non-productive conformation resulted in lower *R*-values (Table 2).

**Table 2:** Data collection and refinement statistics for RT–DNA complexes.

	RT–DNA•(−)-FTC-TP (PDB 6UJX)	RT–DNA•(−)-3TC-TP (PDB 6UJY)	RT–DNA•(+)-FTC-TP (PDB 6UJZ)	RT–DNA•dCTP (PDB 6UIT)
Space group	*C*222_1_	*C*222_1_	*C*222_1_	*C*222_1_
Unit Cell (a, b, c in Å)	169.8, 167.7, 103.3	166.7,169.5,102.8	168.3, 168.8, 102.5	167.5, 167.8, 102.3
Resolution (Å)	50.0–2.65 (2.70–2.65)	50.0–2.60 (2.64-2.60)	50.0–2.55 (2.71-2.55)	50.0–2.8 (2.85–2.80)
No. of reflections	213,787	181,552	280,014	218,437
No. unique	39,743 (3,373)	45,025 (2,238)	47,323 (4,516)	35,132 (3,187)
Redundancy	5.3 (5.2)	4.0 (4.1)	5.8 (5.3)	6.2 (5.9)
*I/σ*	12.8 (1.8)	16.5 (1.8)	12.2 (1.4)	19.3 (2.1)
*R*_merge_^a^	0.11 (0.735)	0.068 (0.497)	0.094 (0.886)	0.081 (0.627)
Completeness (%)	99.9 (100.0)	99.7 (100.0)	99.5 (96.4)	99.6 (99.6)
Refinement statistics				
Resolution (Å)	47.6–2.70	47.5–2.60	47.3–2.55	47.1–2.80
No. reflections (F ≥ 0)	39,631 (3,373)	41,844 (2,904)	47,318 (4,516)	35,062 (3,187)
*R*-factor^b^/*R*-free^b^	19.7/24.6	17.7/23.4	18.5/23.6	18.5/25.6
No. atoms				
Protein	7.774	7709	7795	7704
DNA	820	820	862	842
Ligand/ion/water	56/21/112	54/11/189	28/21/47	28/16/34
Average B factors				
Protein	40.5	45.5	60	44.5
DNA	52.5	56	71	56
Ligand/ion/water	44.5/67.9/34.9	47.6/63.2/42.4	58.3/76.7/56.9	44.4/67.3/33.6
r.m.s. deviations				
Bond lengths (Å)	0.01	0.009	0.01	0.01
Bond angles (°)	1.16	1.18	1.17	1.23
Ramachandran				
Favored/allowed/ outliers (%)	93.7/6.3/0	95.8/4.2/0	95.1/4.5/0.4	94.3/5.7/0
	**M184V-DNA (PDB 6UKO)**	**M184V-DNA•(−)-FTC-TP (PDB 6UIR)**	**M184V-DNA•dCTP (PDB 6UIS)**	
Space Group	*C*222_1_	*C*222_1_	*C*222_1_	
Unit Cell (a, b, c in Å)	168.0, 169.7, 97.4	165.9, 170.1, 103.4	167.6, 169.1, 103.3	
Resolution (Å)	50.0–2.75 (2.80–2.75)	50.0–2.6 (2.64–2.60)	50.0–2.75 (2.80–2.75)	
No. of reflections	146,040	221,174	163, 913	
No. unique	35,981 (3,384)	41,741 (3,432)	37,257 (2,922)	
Redundancy	4.1 (4.1)	5.8 (5.8)	4.3 (4.3)	
*I/σ*	16.1 (3.3)	33.1 (2.5)	25.2 (2.7)	
*R*_merge_^a^	0.083 (0.491)	0.045 (0.575)	0.052 (0.0483)	
Completeness (%)	99.9 (99.9)	99.0 (99.0)	99.6 (100.0)	
Refinement statistics				
Resolution (Å)	47–2.75	47–2.60	47–2.75	
No. reflections (*F* ≥ 0)	35,956 (3,377)	41,702 (3,432)	37,224 (2,922)	
*R*-factor^b^/*R*-free^b^	20.5/26.0	19.6/26.4	19.8/26.8	
No. atoms				
Protein	7.461	7,675	7,728	
DNA	776	776	820	
Ligand/ion/water	0/11/21	28/11/211	28/12/145	
Average B factors				
Protein	45.0	27.0	26.0	
DNA	44.0	38.0	38.5	
Ligand/ion/water	−/67.0/34.9	31.8/39.1/22.9	20.2/47.4/16.9	
r.m.s. deviations				
Bond lengths (Å)	0.01	0.009	0.009	
Bond angles (°)	1.24	1.18	1.17	
Ramachandran				
Favored/allowed/outliers (%)	94.6/5.2/0.2	95.6/4.2/0.2	94.8/5.0/0.2	

^a^*R*_merge_ = [∑h∑i|Ih – Ihi|/∑h∑iIhi] where Ih is the mean of Ihi observations of reflection h. Numbers in parentheses represent highest resolution shell

^b^*R*-factor and *R*-free = ∑||*F*_obs_| − |*F*_calc_||/∑|*F*_obs_| x 100 for 90% of recorded data (*R*-factor) or 10% of data (*R*-free)

The net effect of the non-productive conformation is that the α-phosphate is shifted away from the primer strand, compared to the productive conformation (Fig. 4c). The pivot point for this rotation is through the C5′ carbon that connects the oxathiolane and triphosphates. In the productive conformation, the distance from the C3ʹ in the deoxyribose ring of the last priming nucleotide and the α-phosphate of (−)-FTC-TP is 4.8 Å, while in the second conformation the distance is 7.9 Å. This shift of 3.1 Å of the α-phosphate would likely prevent the proper alignment for nucleophilic attack by the primer 3ʹ-OH and thus, slow the incorporation of (−)-FTC-TP into the DNA primer strand.

Residue R72 has been noted to be involved with proper positioning of the α-phosphate for incorporation and stabilizing the transition state^28^. In this ternary structure, R72 lies across the face of the cytidine base in a near parallel fashion and forms a hydrogen bond with an oxygen directly connected to the α-phosphate (Fig. 4a). The guanidinium group forms a hydrogen bond with Q151 through Nε to help position the side chain of Q151. Due to the positioning of the R72 side chain in the active site, it appears to form a π-stacking interaction with the face of the cytidine base. For (−)-FTC-TP, the 5-fluoro atom in the cytidine ring comes relatively close (3.2 Å) to the R72 nitrogen Nη (Fig. 4a). Since the angle between the fluorine and the nitrogen is not ideal for a hydrogen bond and 5-fluoro is a weak hydrogen bond acceptor, the 5-fluoro likely interacts with R72 through an ion-dipole.

**Fig. 5:**
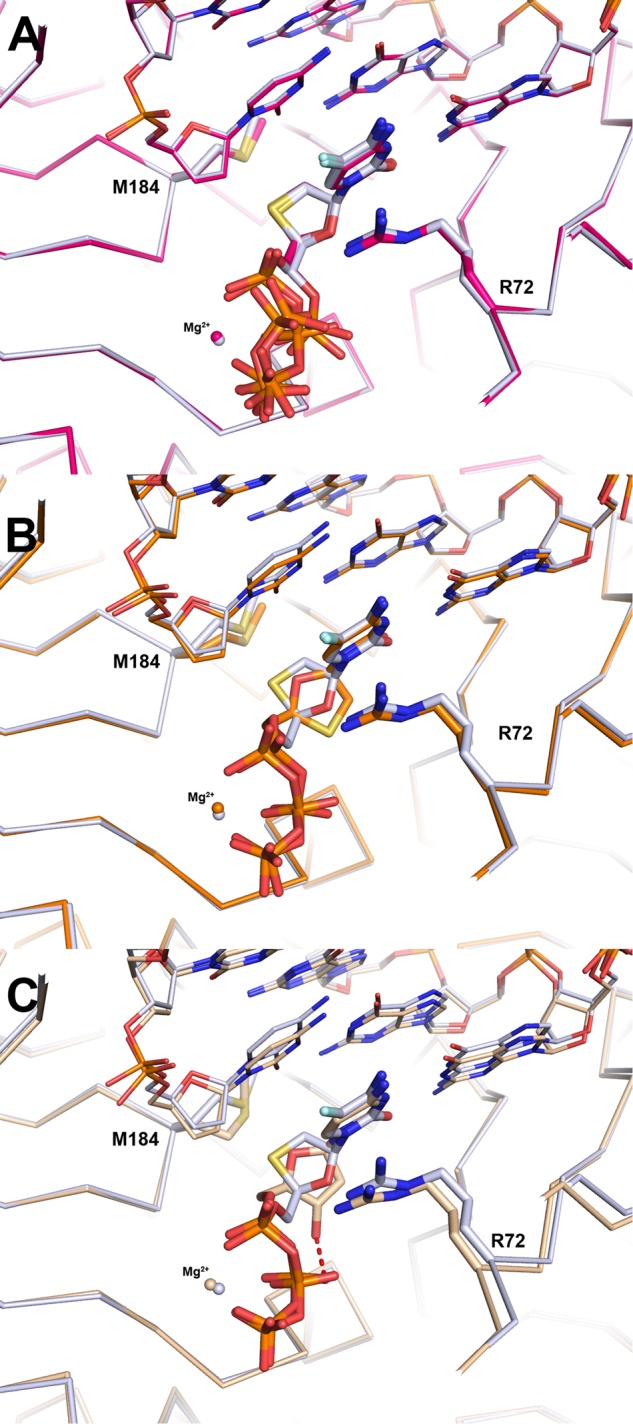
Comparison of (−)-FTC-TP ternary structures to (−)-3TC-TP, (+)-FTC-TP, and dCTP. All alignments are generated comparing residues 107-112 and 151-215 in the p66 subunit. **a** RT–DNA•(−)-FTC-TP (light blue) compared to RT–DNA•(−)-3TC-TP (red). The multiple phosphate positions are drawn for both NRTI triphosphates. **b** RT–DNA•(+)-FTC-TP (orange) places the oxathiolane ring in the normal nucleotide binding position compared to RT–DNA•(−)-FTC-TP (light blue). Only the productive triphosphate conformation in the RT–DNA•(−)-FTC-TP structure is shown for clarity. The positions of the stereocenters overlap and the triphosphate conformations are comparable. **c** Relative to RT–DNA•(−)-FTC-TP (light blue), RT–DNA•dCTP (beige) positions the deoxyribose ring in the canonical binding position for incorporation into DNA. The intramolecular hydrogen bond from the 3’-OH to the β-phosphate oxygen is drawn in a red dashed line. As in panel **b**, only the productive triphosphate conformation is shown for clarity.

The ternary structure RT, DNA, and (−)-3TC-TP (RT–DNA•(−)-3TC-TP) was also crystallized and solved (Fig. 3b). Overall, the binding mode is very similar to the one in RT–DNA•(−)-FTC-TP (Fig. 5a). There is a normal Watson–Crick base pair formed with the template guanine and two conformations are observed for the triphosphates. R72 lies across the cytosine base in the same position as with RT–DNA•(−)-FTC-TP, forming a π-stacking interaction with the base. Since (−)-3TC-TP lacks a 5-fluoro atom in the cytidine base, there is no possibility of forming an ion-dipole with R72. The lack of this interaction is potentially why there is a 2.5-fold higher *K*_d_ value (or a 2.5-fold lower binding affinity) of (−)-3TC-TP over (−)-FTC-TP and 3.7-fold greater incorporation efficiency observed for (−)-FTC-TP over (−)-3TC-TP (Table 1).

### (+)-FTC-TP possesses a similar binding conformation as dCTP

In order to determine the effect of the oxathiolane ring ribose mimic in the context of a *D*-nucleoside, the crystal structure of RT, DNA, and (+)-FTC-TP (RT–DNA•(+)-FTC-TP) was determined. The defined electron density clearly showed the different positions of the oxathiolane atoms between (+)-FTC-TP and (−)-FTC-TP (Figs. 3c and 5b). The position of the oxygen in the oxathiolane ring of the (+) isomer points towards the primer 3′-terminus and the sulfur is positioned near the phosphates (Fig. 3c). There was only one conformation of the triphosphates apparent in the electron density, similar to dCTP (see below) and therefore a productive conformation for incorporation (Fig. 3c, d). The correct positioning of the phosphates appears to be aided by the oxathiolane sulfur mimicking the position of C3ʹ of deoxyribose. An apparent effect of the 3ʹ-sulfur is steric which prevents the α-phosphate from flipping away from the DNA primer into a non-production conformation. Additionally, the 3ʹ-sulfur sits closely (3.3 Å) to the bridging oxygen between α- and β-phosphates (Fig. 3c) which is less than the van der Waals radii of a sulfur and oxygen (1.8 Å + 1.52 Å = 3.32 Å). Our pre-steady-state kinetic parameters for the incorporation of (+)-FTC-TP and (−)-FTC-TP WT RT show similar efficiency (*k*_p_/*K*_d_) values for the two analogs (Table 1). However, the *k*_p_ for (+)-FTC-TP incorporation is improved by 2.4-fold relative to (−)-FTC-TP, possibly due to the phosphates in (+)-FTC-TP being locked into the productive conformation.

In order to compare how the cytosine based NRTIs bind in relation to the natural substrate, the structure of the ternary complex of RT, DNA and dCTP (RT–DNA•dCTP) was determined (Fig. 3d). The binding conformation of dCTP is equivalent to previously reported crystal structures with natural substrates, dTTP (PDB code 1RTD)^29^ and dATP (PDB code 3KK2)^27^. The triphosphates interact with a Mg^2+^ ion with octahedral coordination, as well as R72, K65, and K220. To further help position the phosphates, dCTP forms an intermolecular hydrogen bond (2.8 Å) between its 3ʹ-OH and an oxygen atom from the β-phosphate, thus stabilizing the triphosphate conformation (Fig. 5c). Comparing the central ring, it is striking that the C1ʹ and C4ʹ have nearly perfect alignment between (−)-FTC-TP and dCTP (Fig. 5c). The productive triphosphate binding conformation for RT–DNA•(−)-FTC-TP matches well with the triphosphate conformation in RT–DNA•dCTP. Strikingly, the M184 side chain exists as different rotamers in the WT structures. Although M184 is not in direct contact with the central ring of dCTP or NRTI substrates described here, the orientation of the Cγ atom shifts depending on the incoming nucleotide. Cγ is positioned closer to dCTP and is shifted away from the oxathiolane ring indicating its sensitivity to dCTP vs. a NRTI in WT RT (Fig. 5c).

### Mechanistic basis of M184V resistance to *L*-nucleotide analogs

**Fig. 6:**
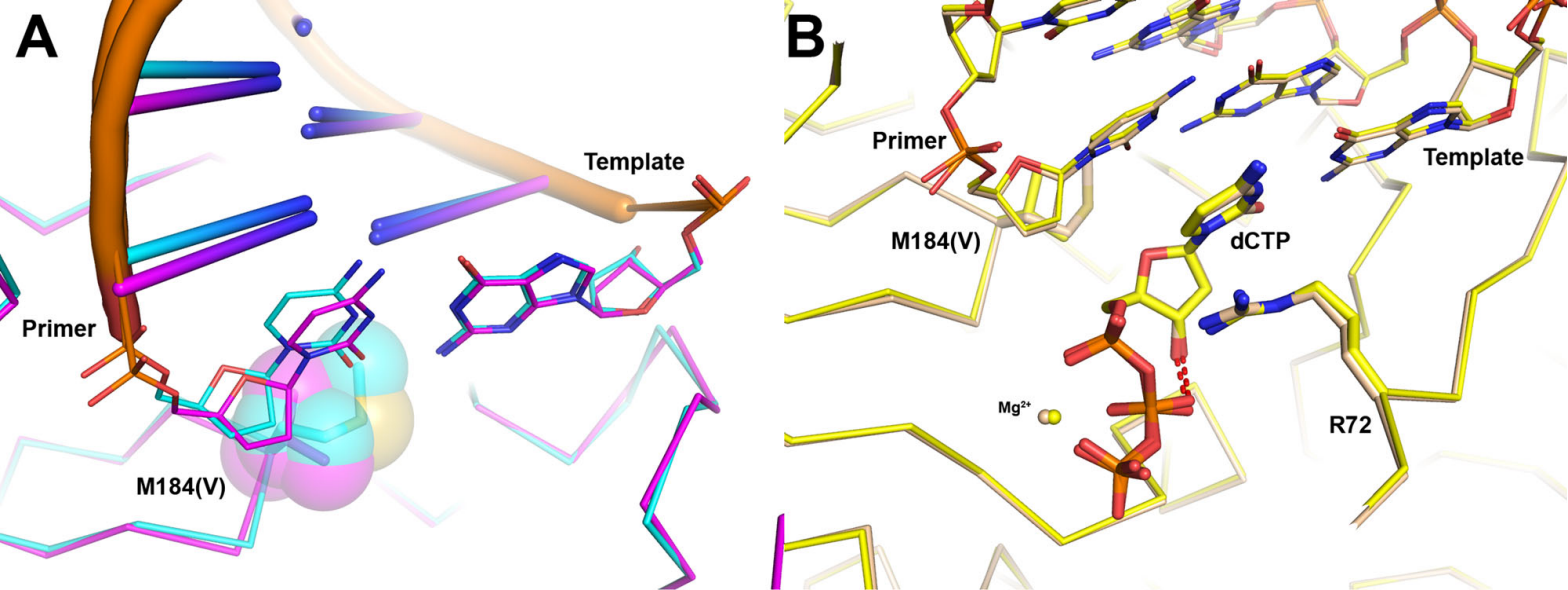
Structures of M184V HIV-1 RT-DNA binary and ternary complex with dCTP. **a** Alignment of the RT–DNA (PDB code 3KJV, cyan) and M184V-DNA (PDB code 6UKO, magenta) binary complexes. The M184V mutation is positioned in the P-site (priming site) directly below the primer 3′-terminal nucleotide. Spheres are drawn to illustrate how valine fills out the pocket and limits possible rotation of the side chain. Neighboring amino acids are not affected by the mutation. **b** Overlay of the ternary structures of RT–DNA•dCTP and M184V-DNA•dCTP. There is no steric clash between M184V and dCTP. dCTP is positioned for incorporation (productive binding conformation) whether bound to WT RT or M184V.

The M184V mutation was introduced into both the p66 and p51 subunits of RT (Methods) and the crystal structure of the binary complex of RT M184V and DNA (M184V-DNA) was solved (Fig. 6a). The electron density for the valine residue was clearly visible and the atom positions were assigned for the side chain in both subunits. Comparing M184V-DNA to RT–DNA (PDB code 3KJV) in the same crystallography system, i.e. space group and DNA sequence, the V184 is orientated in the P-site under the deoxyribose ring of the primer-terminal base and shows no apparent change to the conformation of other amino acids in the polymerase active site (Fig. 6a). Alignment of RT–DNA and M184V-DNA structures resulted in an RMSD of 0.39 Å. There appears to be a small shift in the primer 3′-terminal nucleotide in response to the mutation which was also observed in the previous binary structure of M184I RT bound to DNA^30^.

**Fig. 7:**
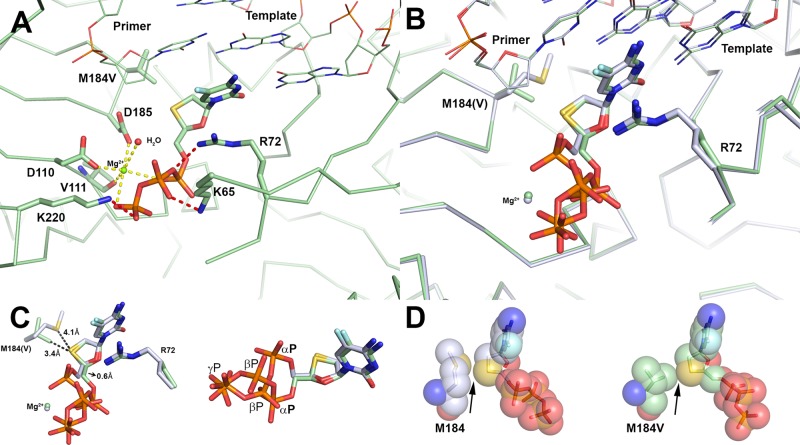
Ternary crystal structure of M184V-DNA bound to (−)-FTC-TP. **a** The coordination of the Mg^2+^ has an octahedral arrangement, however, the β-phosphate is not participating in coordination and the void is filled by one water molecule. **b** Comparing to the WT structure (light blue), the oxathiolane ring has a minor shift with the sulfur moving 0.3 Å. This movement translates to the α-phosphate shifting into an alternate conformation too far (7.9 Å from priming C3ʹ-deoxyribose) to be efficiently incorporated into the primer strand. **c** Alternate view of (−)-FTC-TP binding conformation with WT and M184V structures. **d** Spheres drawn around M184(V) side chain and (−)-FTC-TP illustrating the close proximity between the oxathiolane sulfur and residue 184. In the WT structure, there is room between methionine side chain and the oxathiolane ring, however, with the valine mutation, there is no extra space.

As with the above-mentioned WT structures, the same procedure (Methods) was used to generate the ternary structure of M184V, DNA, and (−)-FTC-TP (M184V-DNA•(−)-FTC-TP) (Fig. 3e). The resolution (2.75 Å, Table 2) and quality are comparable between the structures in Fig. 3. Overall, the positions of the cytidine and oxathiolane are similar as in the RT–DNA•(−)-FTC-TP structure (Fig. 7b). There is only one conformation of the triphosphates observed (Fig. 7a) which matches the non-productive conformation observed in RT–DNA•(−)-FTC-TP and RT–DNA•(−)-3TC-TP. As with the RT–DNA•(−)-FTC-TP, there is a Mg^2+^ ion with an octahedral ligand coordination sphere observed; however the Mg^2+^ interacts with the α- and γ-phosphates and not the β-phosphate (Fig. 7a). Because the β-phosphate is shifted and not interacting with the Mg^2+^, a water molecule was observed completing the coordination sphere (Fig. 7a). Even though Mg^2+^ has very stringent requirements for a coordination sphere (i.e. lengths and angles), it is still able coordinate the triphosphates in this non-productive conformation. The lack of change in the Mg^2+^ location is perhaps due to the protein atoms not moving, which provides an anchor point to coordinate the flexible triphosphates.

Examining the van der Waals radius of V184, it is clear that there is a direct interaction with the branched side chain and the sulfur in the oxathiolane ring of (−)-FTC-TP (Fig. 7d). For the RT–DNA•(−)-FTC-TP complex, the closest distance of the oxathiolane ring to M184 was 4.1 Å, compared to 3.4 Å for the valine side chain (Fig. 7c). Structure alignment through residues 107–112 and 151–215 in the p66 subunit between the WT and M184V structures with (−)-FTC-TP shows that the oxathiolane sulfur is shifted away from residue V184 by 0.3 Å compared to M184. A concerted movement is observed in (−)-FTC-TP that results in the C5´ carbon, which is the pivot point of the different triphosphate orientations, further shifting by 0.6 Å. This perhaps has a larger effect on the triphosphate orientation. Although the calculated maximum likelihood coordinate error reported in Phenix refinement is 0.33 Å for the WT and M184V (−)-FTC-TP structures, this shift is propagated to cause other atoms of (−)-FTC-TP to move more than 0.33 Å from the WT structure. The combination of a steric shift and altered triphosphate orientation likely results in reduced binding affinity of (−)-FTC-TP (280-fold higher *K*_d_ in Table 1) with M184V than with WT RT.

As with WT RT, the crystal structure of M184V-DNA bound to dCTP (M184V-DNA•dCTP) was determined (Fig. 3f). The overall binding conformation is nearly identical for RT–DNA•dCTP and M184V-DNA•dCTP (Fig. 6b), and there is no apparent steric clash with the valine side chain. Our pre-steady-state kinetic data support that M184V does not discriminate against dCTP based on similar kinetic parameters measured for both WT and M184V (Table 1). Analysis of various RT–DNA ternary complexes in the PDB^27,29,31,32^ reveals a wide range of M184 conformations. Similarly, with our solved WT structures of (−)-FTC-TP, (−)-3TC-TP, (+)-FTC-TP, the methionine shifts away from the oxathiolane sulfur compared to dCTP (Fig. 5c). Remarkably, the γC of M184 in the RT–DNA•dCTP structure mimics the branched carbon of valine, suggesting that it has some role in stabilizing dCTP binding (Fig. 6b). M184 shifts upon NRTI binding, whereas V184 remains static and keeps (−)-FTC-TP or (−)-3TC-TP in a non-productive conformation, thus likely preventing their tight binding and efficient incorporation as quantified through our pre-steady-state kinetic parameters (Table 1).

## Discussion

Often the best candidate for drug development balances important properties such as potency, metabolic stability, and limited off-target toxicity. FTC and 3TC represent a distinct class of nucleoside antiviral drugs which contain an unnatural (−)-β-L stereochemistry for a deoxyribose sugar analog. The fact that HIV-1 RT and HBV polymerase can recognize these drugs and effectively incorporate them is remarkable. Likewise, the fact that human kinases can utilize the free hydroxyl (prodrug) form to convert them into the active triphosphate metabolite is equally surprising. The crystal structures of RT–DNA•(−)-FTC-TP and RT–DNA•(−)-3TC-TP presented here provide a structural basis for binding of the (−)-β-L oxathiolane ring to HIV-1 RT. The L configuration is well tolerated by RT with no steric clash from protein residues or DNA to effectively discriminate against it. There are two distinct conformations of the incoming triphosphates of (−)-FTC-TP and (−)-3TC-TP. One conformation placed the triphosphate chain in an orientation consistent with RT–DNA•dCTP and was presumably a productive state for incorporation. The other non-productive conformation of the triphosphate chain is possibly due to a lack of steric hindrance afforded by the part of the oxathiolane ring furthest from the primer, or the removal of an intramolecular 3´OH to β-phosphate bond that is found in RT-bound natural dNTPs. The tighter binding of the oxathiolane ring can also be attributed to greater hydrophobic contact with Y115 due to the larger sulfur atom (Fig. 4a).

M184V is the primary resistance mutation generated during HIV treatment with FTC and 3TC^6–8^. Generally, M184I precedes the M184V mutation and can arise through a single nucleotide change from G to A. Hypermutation at this position has been reported for HIV^33^. However, M184V requires a two nucleotide change and in some cases has been shown to mutate directly from WT and not through a progression of I to V^10,33^. M184V eventually outcompetes M184I based on higher RT polymerase activity and better processivity^34^. The M184V mutant confers some of the highest resistance as a single mutation against an NRTI with up to >300-fold reduction in potency in enzyme-based assays and >500-fold reduction in cell-based assays for FTC and 3TC^7,18^.

A prior binary crystal structure of M184I RT–DNA predicted a steric clash with the oxathiolane ring. Additionally it was noted that there was movement of the primer terminus for which M184I or V is directly below^30^. Thus, it was hypothesized that the movement of the primer terminus to a less reactive position was the cause of the reduced reactivity of the mutant toward (−)-FTC-TP and the cause of the resistance. In the M184V binary structure reported here, the position of the primer terminus does not appear to have a large effect on the mechanism of resistance. In the binary structure of M184V-DNA, there is a shift in the DNA primer compared to RT–DNA (Fig. 6a). However, in both M184V-DNA•(−)-FTC-TP and M184V-DNA•dCTP the primer shifts back to a position comparable to WT (Fig. 3). Therefore it appears unlikely that improper positioning of the primer terminus has an effect in resistance to NRTIs.

The WT structures showed two distinct positions of the Cγ atom of M184. For RT–DNA•dCTP, Cγ is positioned closer to the ribose ring. For RT–DNA•(−)-FTC-TP, RT–DNA•(−)-3TC-TP, and RT–DNA•(+)-FTC-TP the Cγ atom is flipped away from the oxathiolane ring (Fig. 5a–c). Comparing the position of Cγ in RT–DNA•(−)-FTC-TP to RT–DNA•dCTP, the atom moves 1.8 Å (Fig. 5c). Comparatively, the Cγ1 and Cγ2 atoms of V184 occupy very similar positions to the two rotamer positions of M184 Cγ in the WT structures (Fig. 6b). Valine therefore reduces the conformational flexibility apparent with M184 which aids in the binding of these NRTIs to WT RT. The net effect of the M184V mutation produces a steric hindrance by directly contacting the sulfur atom in the oxathiolane ring (Fig. 7d). During (−)-FTC-TP and (−)-3TC-TP incorporation by WT RT, their triphosphates are found in productive and nonproductive conformations (Fig. 5a); however, the M184V mutation shifts the conformation equilibrium towards the catalytically ineffective state (Fig. 7c). This is consistent with measured *K*_d_ of nucleotide binding which increased from 0.10 and 0.25 µM with WT HIV-1 RT to 28 and 53 µM with M184V for (−)-FTC-TP and (−)-3TC-TP, respectively, and with measured substrate specificity (*k*_p_/*K*_d_) which decreased from 1.0 and 0.27 µM^−1^ s^−1^ to 2.6 × 10^−3^ and 9.4 × 10^−4^ µM^−1^ s^−1^ for (−)-FTC-TP and (−)-3TC-TP, respectively (Table 1). Taken together, our pre-steady-state kinetic data in combination with our crystal structures support the mechanism whereby the M184V mutation confers resistance via primarily affecting the binding and proper orientation of (−)-FTC-TP and (−)-3TC-TP within the active site of HIV-1 RT.

Recently, ternary crystal structures were reported of human mitochondrial DNA polymerase γ, DNA, and FTC-TP or 3TC-TP^21^. Based on a structural comparison to HIV-1 RT, it was postulated that there could be a hydrogen bond formed between R72 in RT and the 5-fluoro of (−)-FTC-TP. The structures reported here, demonstrate a likely interaction between the 5-fluoro and R72; however, the contact appears to be best characterized as an ion-dipole interaction. The angle between the guanidinium nitrogen of R72 and 5-fluoro is not ideal for hydrogen bond formation. Consistently, our pre-steady-state kinetic data indicate a 2.5-fold tighter binding for (−)-FTC-TP (*K*_d_ = 0.10 µM) than (−)-3TC-TP (*K*_d_ = 0.25 µM, Table 1), likely caused by the ion-dipole interaction between R72 and the 5-fluoro of (−)-FTC-TP. Interestingly, R72 is one of the few invariant residues found in RT which does not mutate in response to drug treatment^35^. In the case of HIV-1 RT, R72 plays a role in tighter binding of (−)-FTC-TP over (−)-3TC-TP.

Other crystal structures of (−)-FTC-TP have been reported in ternary complexes with human DNA polymerases λ (Polλ)^20^ and β (Polβ)^26^ as well as *S. solfataricus* Dpo4^25^. Both Polλ and Polβ are involved with DNA repair and are implicated in off-target toxicity by NRTIs^23^. Polλ was crystallized with a single nucleotide gapped DNA substrate and (−)-FTC-TP (PDB code 4K4I). Interestingly, when bound in the active site of Polλ (Complexes A and E in the crystal structure), (−)-FTC-TP exhibits a similar binding conformation as when bound to RT. For the other two complexes of Polλ in the asymmetric unit, R517 surprisingly used its side chain to coordinate the cytidine base through two hydrogen bonds^20^. No comparable residue exists in RT and only normal Watson–Crick base pairing of cytosine to guanine was observed with RT (Fig. 3). Unexpectedly, the pre-catalytic ternary structure of Polβ, a single nucleotide gapped DNA substrate, and (−)-FTC-TP (PDB code 5U2T) shows that Polβ, a sequence and structure homolog of Polλ, bound (−)-FTC-TP with Watson–Crick base pairs and productive triphosphate conformations, but with accumulation of several active site rearrangements that led to decreased nucleotide binding affinity and incorporation rate^26^. We also reported the ternary structure of Dpo4, dsDNA, and (−)-FTC-PPNP, a non-hydrolyzable triphosphate analog where a nitrogen replaced the bridging oxygen between the β- and γ-phosphates (PDB code 4QW9). Multiple conformations of the phosphates were also observed in this Dpo4 ternary structure^25^ which is consistent with the observed triphosphate conformations observed in our RT–DNA•(−)-FTC-TP and RT–DNA•(−)-3TC-TP structures reported in Fig. 5a. Taken together, the aforementioned DNA polymerases and HIV-1 RT bind and incorporate (−)-FTC-PPNP through unique structural mechanisms^26^.

In addition to FTC and 3TC, other drugs with similar stereoisomer chemistry have progressed into human clinical trials. Racivir was developed as a 50/50 racemic mixture of (+)-FTC and (−)-FTC and was tested in Phase II clinical trials (NCT00121979). It was reported that (+)-FTC selects for T215Y as opposed to M184V in cell culture^36^. Although M184V still conferred 30-fold selection against (+)-FTC-TP (Table 1), it seems likely that (+)-FTC may not choose for this mutation since the oxathiolane ring is turned away from M184 (Fig. 3c). Telbivudine, the *L*-nucleoside analog of thymidine, has been successfully developed to treat HBV^37^. Reportedly, this drug does not inhibit HIV^37^. Apricitabine (AVX754, SPD754) is also a cytidine analog containing an oxathiolane ring. This oxathiolane has natural D stereochemistry but the position of the oxygen and sulfur atoms are switched in comparison to (+)-FTC and (+)-3TC^38^. This drug reportedly entered Phase IIb/III clinical trials but further development was halted (NCT00612898).

Nucleoside analogs are powerful antiviral drugs and the development of new NRTIs are constantly needed to combat the emergence of drug resistance in addition to the threat of emerging epidemics such as the Zika virus outbreak in 2016. Understanding the basis of resistance that arises from viral mutation is paramount for developing efficacious drugs therapies. In the case of (−)-FTC-TP and (−)-3TC-TP, M184V causes resistance by sterically hindering the oxathiolane ring and pushing the phosphates away from the primer strand DNA, thus locking them in a non-productive conformation (Fig. 7). One design strategy to avoid improper phosphate positioning would be to adjust the steric bulk around the central ring to prevent the phosphates from flipping to a non-productive state, thus maintaining the flexibility of the methionine at position 184. This is akin to the (+)-β-*D* stereochemistry which only allows the phosphates to exist in a productive conformation (Fig. 3c). This could potentially be achieved with substitutions to the oxathiolane ring that rigidify the ring but would still be accepted in the NRTI binding pocket. In addition, further optimization of NRTI interactions with R72 could increase the potency against HIV-1 RT as this residue is essential for polymerase function and not readily mutable. Identifying analogous residues to R72 in other viral polymerases could also be a fruitful avenue for improving the efficacy of drug therapies against selected viruses.

FTC and 3TC have been key components of HAART and HIV treatment. The structures reported here reveal how HIV-1 RT recognizes the unnatural (−)-β-*L* stereochemistry. In addition, our crystal structures of HIV-1 RT M184V show how this mutation could potentially confer resistance to (−)-FTC-TP and (−)-3TC-TP. Furthermore, our combined structural and kinetic analysis will aid in rational drug design to combat the HIV pandemic.

## Methods

### Expression and purification of the RT–DNA complex

HIV-1 RT p66 and p51 subunits were expressed and purified as described previously^27,39^. Site-directed mutagenesis to generate the M184V the mutation in both p66 and p51 subunits was performed using the QuickChange II kit (Stratagene, La Jolla, CA) according to manufacturer’s protocol. The primer and template oligonucleotides were purchased from TriLink Biotechnologies (San Diego, CA) and resuspended in 1 mM HEPES pH 7.5. The 26-mer DNA template (5ʹ-ATGGGGGGCGCCCGAACAGGGACTGT-3ʹ) was annealed to a dideoxy terminated 18-mer nucleotide primer (5ʹ-GTCCCTGTTCGGXCGCCC_dd_-3ʹ). The X in the primer sequence represents *N*^2^-cystamine-deoxyguanosine which covalently cross-links to Q258C in the p66 subunit. The method of covalently tethering HIV-1 RT dsDNA to generate the RT–DNA complex has been formally described^27,29,40^. To form the covalent complex, RT and dsDNA substrate were incubated together at 25 µM and 50 µM, respectively at room temperature for approximately 18 h. The progress of the tethering reaction was monitored by the observation of an increase in the molecular weight of the p66 subunit as judged by non-reducing SDS-PAGE. Covalently linked RT–DNA complexes were purified^27^ and mass spectrometry performed to analyze the purified sample confirming that a homogeneous heterodimer consisting of p66 tethered to DNA and p51 subunit existed.

### Measurement of pre-steady-state kinetic parameters

HIV-1 RT (120 nM) and 5ʹ-[^32^P]-labeled 18/26-mer DNA (30 nM; DNA sequence shown above the 18mer primer without chemical modification) were pre-incubated at 37 °C for 5 min in reaction buffer containing 50 mM Tris-HCl (pH, 7.9), 10 mM MgCl_2_, 50 mM NaCl, 0.1 mM EDTA, 10% glycerol, 5 mM DTT, and 0.1 µg/mL BSA (all concentrations are final upon mixing). The reactions were initiated by rapidly mixing with varying concentrations of dCTP, FTC-TP, or 3TC-TP on a rapid chemical-quench flow apparatus (KinTek) and quenched with 0.37 M EDTA at various times. The DNA products were analyzed by sequencing gel electrophoresis (17% polyacrylamide, 8 M Urea), and quantitated using a Typhoon Trio (GE Healthcare) and ImageQuant software (Molecular Dynamics). The kinetic data were fit using non-linear regression software KaleidaGraph (Synergy) to a single-exponential equation

$$\left[ {{\mathrm{Product}}} \right] = A\left[ {1 - {\mathrm{exp}}\left( { - k_{{\mathrm{obs}}}t} \right)} \right],$$

where *A* and *k*_obs_ represent the reaction amplitude and observed nucleotide incorporation rate constant, respectively. Values for *k*_obs_ were then plotted against the respective dNTP (or an NRTI triphosphate) concentration and each plot was fit to a hyperbolic equation

$$k_{{\mathrm{obs}}} = k_{\mathrm{p}}\left[ {{\mathrm{dNTP}}} \right]/\left( {K_{\mathrm{d}} + \left[ {{\mathrm{dNTP}}} \right]} \right),$$

where *k*_p_ is the maximal nucleotide incorporation rate constant and *K*_d_ is the apparent equilibrium dissociation constant for the binding of dNTP (or an NRTI triphosphate) to the RT and DNA complex. Each set of kinetic parameters (*k*_p_, *K*_d_, *k*_p_/*K*_d_) are derived from 8 individual experiments where the concentration of nucleotide is variable (i.e. 8 distinct concentrations of dCTP or NRTI). All reported error values are derived from data fitting using non-linear regression software KaleidaGraph (Synergy). For kinetic experiments, we did not cross-link RT and DNA.

### Crystallization and data collection

Crystals of the binary WT and M184V RT–DNA complex were grown by hanging drop vapor diffusion over a mother liquor containing 2–4% PEG 4000, 100 mM MES (pH = 6.0), and 10 mM MgSO_4_ at either 4 or 20 °C. Equal parts of protein and reservoir solution were mixed to produce 4 µl drops. To obtain ternary complexes with FTC-TP, 3TC-TP or dCTP, binary RT–DNA crystals were placed into a mother liquor of 36% PEG 4000, 6% glycerol, 100 mM MES (pH = 6.0), and 10 mM MgSO_4_ with 0.5 mM of the NRTI triphopshate, or dCTP for 5–18 h. Prior to data collection, crystals were moved from the above buffer and flash-cooled in a bath of liquid nitrogen. All X-ray diffraction data were collected at The Advanced Light Source (Table 2) at a temperature of 100 K and processed with HKL2000^41^ or XDS^42^.

### Structure determination and refinement

Molecular replacement was performed by the refinement package Phenix^43^ using the starting model PDB code 3KK1^27^ (ternary complex) for WT and M184V structures. The molecular replacement for the structure of binary M184V RT–DNA was determined using PDB code 3KJV^27^ (binary complex) as the starting model. Rigid body refinement, simulated annealing, energy minimization, and B-factor refinement were performed with Phenix. Model building was carried out by the molecular graphics program Coot^44^.

### Reporting summary

Further information on research design is available in the Nature Research Reporting Summary linked to this article.

## Supplementary information

Reporting Summary

## Data Availability

Atomic coordinates and structure factors for the reported crystal structures have been deposited with the Protein Data Bank. Accession numbers are 6UJX, 6UJY, 6UJZ, 6UIT, 6UKO, 6UIR, and 6UIS for WT RT–DNA with (−)-FTC-TP, (−)-3TC-TP, (+)-FTC-TP, and dCTP, binary M184V RT–DNA, M184V RT–DNA with (−)-FTC-TP, and M184V RT–DNA with dCTP, respectively.
